# King in Manchester

**Published:** 1920-04-03

**Authors:** 


**THE PUBLIC WELL-BEING—*(continued)*.**

### KING IN MANCHESTER.

### Good Work Among the Disabled.

The King had a most loyal and enthusiastic welcome in Manchester on Saturday, and spent what he himself described as a most enjoyable day. The main object of his visit was to see what Manchester is doing in the reconstruction of the lives and limbs of men disabled in the war. He went first to the Ministry of Labour's Training Centre in the City Hall, where men, prevented from following their pre-war occupations, are taught in fifteen different trades. The men here, varying in age from forty-five to young lads, number about 320, and during training receive allowances—the single man 40s. a week, and the married men 10s. extra for a wife, 7s. for the first child, and 6s. for each subsequent child. Already 112 men have been well placed, and failures have been remarkably few. His Majesty talked freely with many of the workers, and discussed their wounds and war services.

He also visited Grangethorpe Hospital, some distance out of the city, where orthopædic and other cases are treated under the direction of the Ministry of Pensions. It is said to be one of the best-equipped institutions of its kind in the kingdom. Over two and a-half thousand men are receiving treatment, and what is done here follows the lines suggested the other day at a meeting of the Medical Society of London regarding the re-education of those who have suffered from amputations.

Every day 1,457 pensioners attend for treatment and dressings, while almost every one of the 300 in-patients receives massage treatment. There are thirty masseuses and one masseur to attend to the patients.

The King saw the men employed in the curative workshops, which are one of the most interesting features of the hospital. It has been found that where a man has had too ample leisure to brood over his disability his case has not been as amenable to scientific treatment as it is desirable that it should be. A partial removal of this psychological barrier to a complete cure has been effected by the

THE PUBLIC WELL-BEING—(*continued*).

workshops, which, in engaging and interesting the men in some new occupation, stimulate the mind to healthy activity and brace the body anew. Attendance at the workshops is voluntary, but a large number of the patients put in several hours there every day. A gymnasium is a necessary part of the hospital's equipment, and a new recreation hut has just been given by the British Red Cross Society, and will be staffed by the Fallowfield and Rusholme Detachment of the B.R.C.S. The medical superintendent of the hospital is Dr. C. A. Lees, C.B.E., late colonel of the 2nd London General Hospital, Chelsea, and the matron is Miss A. Woodhouse, R.R.C.

While at the hospital His Majesty invested three sisters and a staff nurse with the R.R.C. (second class) for service at home and abroad. The names of those decorated are Sister Grace M. Hooper and Staff Nurse Sheila O'Riorden, who served abroad; Sister Elizabeth Salisbury, who was in charge of the hospital at Alymer Park, Levenshulme, Manchester; and Sister Agnes M. Moffat, who was at Ducie Avenue Hospital, Manchester.

